# Frontier and hotspot evolution in cerebrotendinous xanthomatosis: a bibliometric analysis from 1993 to 2023

**DOI:** 10.3389/fneur.2024.1371375

**Published:** 2024-07-26

**Authors:** Fei Luo, Yali Ding, Shanyun Zhang, Juanjuan Diao, Bin Yuan

**Affiliations:** ^1^Department of Pediatrics, Affiliated Hospital of Nanjing University of Chinese Medicine, Nanjing, China; ^2^Department of Pediatrics, Nanjing Gaochun Traditional Chinese Medicine Hospital, Nanjing, China; ^3^Department of Pediatrics, Haining Hospital of Chinese Medicine, Haining, China; ^4^Department of Pediatrics, Affiliated Hospital of Shandong University of Traditional Chinese Medicine, Jinan, China

**Keywords:** bibliometric analysis, cerebrotendinous xanthomatosis, CiteSpace, VOSviewer, Web of Science

## Abstract

**Background:**

Cerebrotendinous xanthomatosis (CTX) is an autosomal recessive disease associated with lipid metabolic disorders. Because of its clinical diversity and rarity, the diagnosis is often unclear. However, there is still a lack of reports on bibliometric analysis of CTX. The aim of this study was to assess the progress and research developments of CTX over the past three decades, identify emerging trends, and establish novel directions for future research.

**Methods:**

The eligible literature were screened from the Web of Science Core Collection (WoSCC) database. The annual publication, countries, institutions, authors, journals, keywords and references were visually analyzed by Microsoft Excel 2019, CiteSpace 6.2.R4, VOSviewer 1.6.18 and online bibliometrics website (https://bibliometric.com/).

**Results:**

A total of 561 publications from WoSCC were included in this study. The United States is the country with the largest number of publications, and Karolinska Institutet is the institution with the largest number of publications. Björkhem I. ranks as the most published and cited author in the last three decades. Journal of Lipid Research is the most widely published and cited journal. The strongest burst of keywords is “diagnosis.”

**Conclusion:**

Unraveling the pathogenesis of CTX and improving its diagnosis and treatment continue to be critical challenges that require urgent attention. Future research endeavors will be centered on enhancing the efficiency and accuracy of early diagnosis and intervention.

## 1 Introduction

Cerebrotendinous xanthomatosis (CTX, OMIM 213700) is a rare autosomal recessive disorder first delineated in 1937 by Van Bogaert et al. (1), who described two cousins exhibiting chronic progressive neurological symptoms, one with concurrent cataracts and tendon xanthomas, classified as a systemic cerebral cholesterol storage disease. CTX is precipitated by mutations in the sterol 27-hydroxylase (*CYP27A1*) gene, characterized by the accumulation of abnormal sterols and bile acid precursors in various tissues (2, 3). Elevated cholestanol levels in CTX patients were first documented by Menkes et al. (4) in 1968. In 1971, Salen (5) subsequently elucidated diminished bile acid (predominantly CDCA) excretion in patients, contributing to cholesterol and cholestanol deposition in tendon xanthomas. The biochemical foundation of CTX, linked to sterol 27-hydroxylase deficiency, was definitively established by Oftebro et al. (6) in 1981. The first report of pathogenic mutations in the *CYP27A1* gene by Cali et al. (7) in 1991 advanced the understanding of the molecular genetics underpinning CTX. Reports indicate that the prevalence of CTX is highest among Asian populations, with rates ranging from 1 in 44,407 to 1 in 93,084. Conversely, the lowest prevalence is found among Finns, at a rate of 1 in 3,388,767. The prevalence among Europeans, Americans, and Africans/African-Americans is moderate, estimated to be between 1 in 70,795 and 1 in 233,597 (8). It can be manifested as neonatal cholestatic jaundice (9). Common symptoms of the condition encompass tendon xanthomas, osteoporosis, coronary heart disease, and a range of progressive neuropsychiatric issues. Neurological disruptions often include peripheral neuropathy, pyramidal signs like spastic paraparesis, cerebellar ataxia, cognitive difficulties, and various movement disorders. During childhood and adolescence, the predominant signs are typically bilateral juvenile cataracts, persistent diarrhea, and intellectual challenges (10, 11). The diagnosis is challenging and often unclear due to the rarity of the disease and its various non-specific symptoms. Current diagnosis primarily relies on clinical presentation, biochemical assays, and confirmatory genetic analysis, with neuroimaging serving as an adjunctive diagnostic tool. Treatment predominantly involves bile acid replacement therapy, such as chenodeoxycholic acid (CDCA).

CiteSpace (12) and VOSviewer (13) are bibliometrics softwares, which can analyze the research trends and frontier hotspots in a certain field through the integration of keywords, authors, countries, institutions and references of related publications. Web of science is the most authoritative citation database with the largest number of disciplines in the world, and focuses on natural sciences (14). Compared with Scopus, WOS, it is more accurate and periodic (15). Compared with the lack of citation information in PubMed, the publication information of WOS is more comprehensive (16, 17).

In this study, based on the Web of Science Core Collection (WoSCC) database, the literature related to CTX in the past 30 years was statistically analyzed and visual map was drawn to clarify the research trend and frontier hotspots of CTX.

## 2 Materials and methods

### 2.1 Data selection

We searched CTX literature at Web of Science Core Collection (WoSCC) on December 31, 2023. The following search strategies were used: TS = (cerebrotendinous xanthomatosis) AND DT = (Article OR Review) AND language = (English) AND PY = (1993.01.01–2023.12.31).

**Figure 1:**
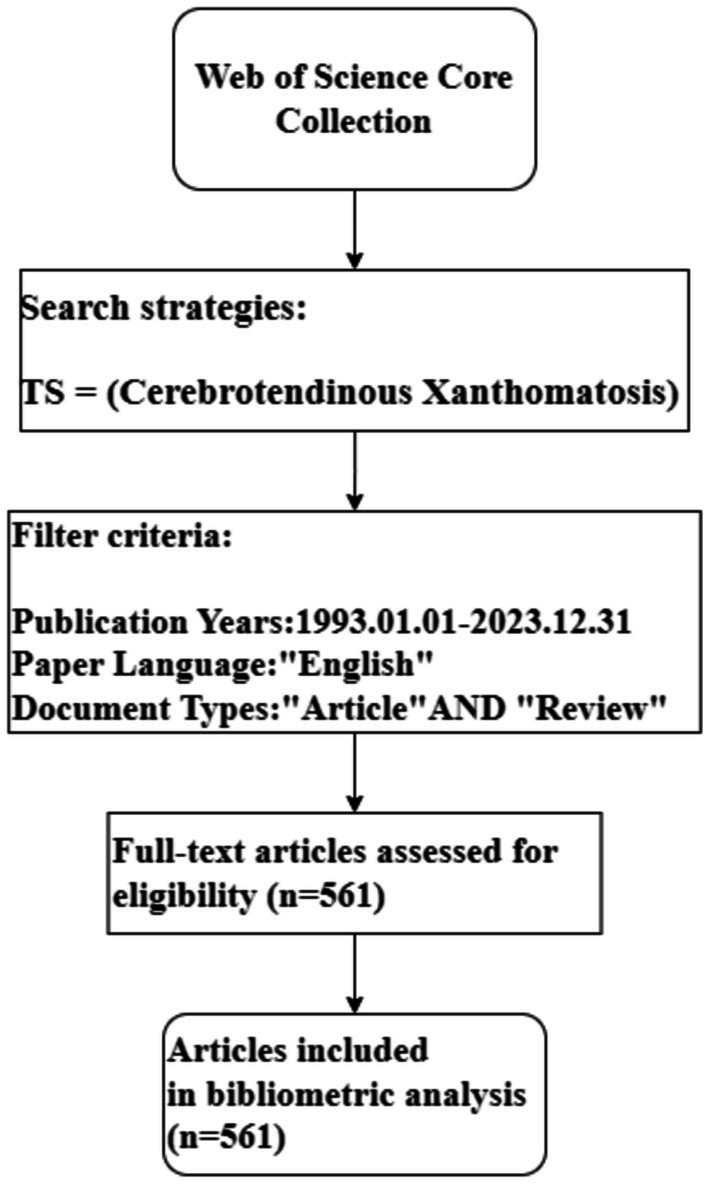
Flowchart of literature search.

A total of 561 publications met the retrieval requirements, and the fully recorded and cited references of the literature are derived in plain text format. The flowchart of the literature search is depicted in Figure 1.

### 2.2 Data analysis

Key information such as title, author, country, institution, keyword, and the year of publication were extracted from articles that met the criteria. Microsoft Excel 2019, CiteSpace 6.2.R4, VOSviewer 1.6.18 and online bibliometrics website^1^ were used to process the above data.

## 3 Results

### 3.1 Trends in publication outputs

**Figure 2:**
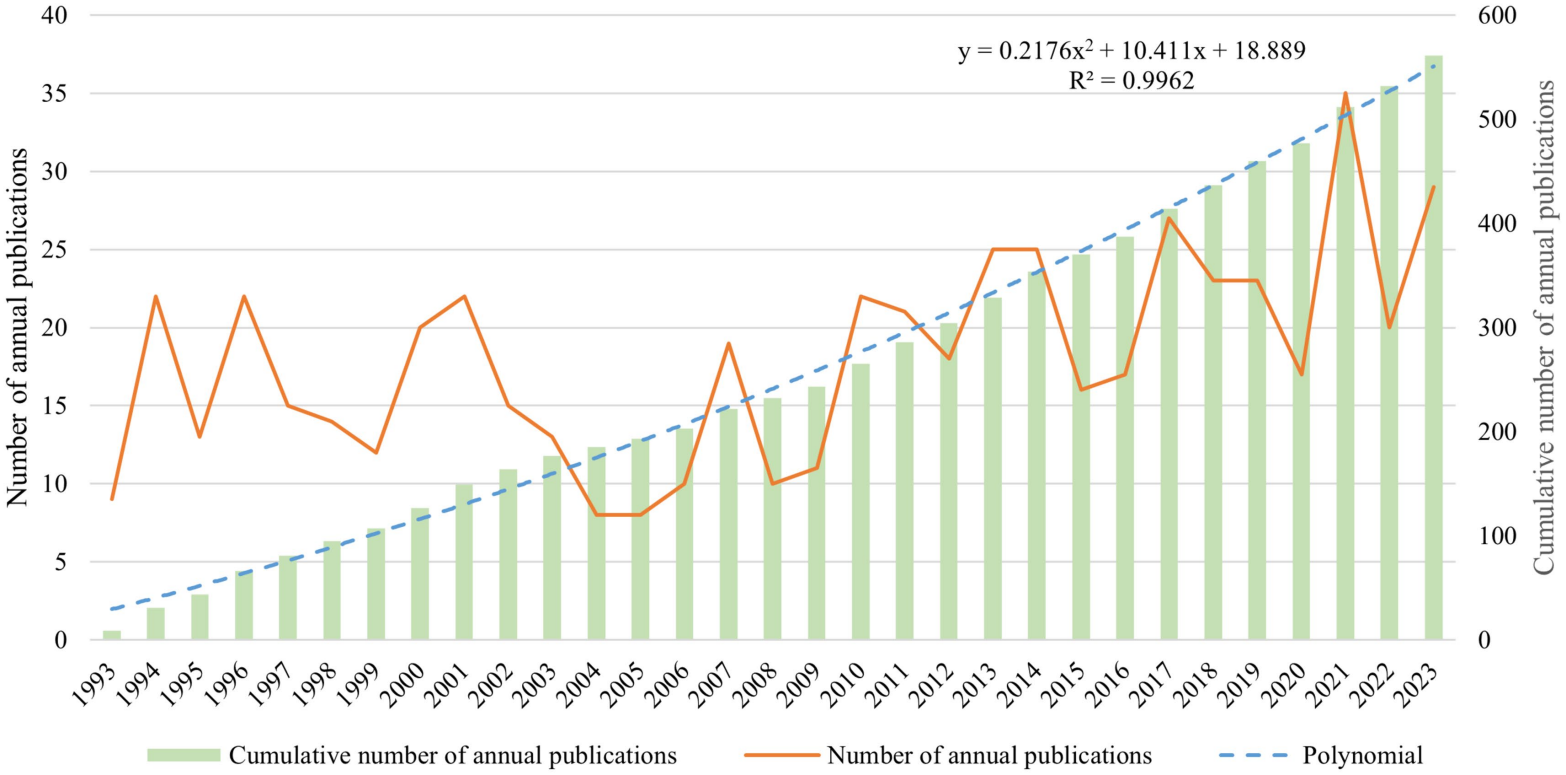
Trend of publications in the field of CTX (1993–2023).

Figure 2 depicts the annual number of CTX-related publications. Over the past 30 years, the cumulative yearly total shown an overall upward trend, despite significant fluctuations in the annual number of papers published each year. In order to understand the trend of CTX research, the polynomial function *Y* = 0.2176*X*^2^ + 10.411*X* + 18.889 (*R*^2^ = 0.9962, *X* is the year, *Y* is the annual cumulative number of articles) was constructed. And 2021 is the peak year for publication, with 35 articles.

### 3.2 Analysis of countries and institutions

**Figure 3:**
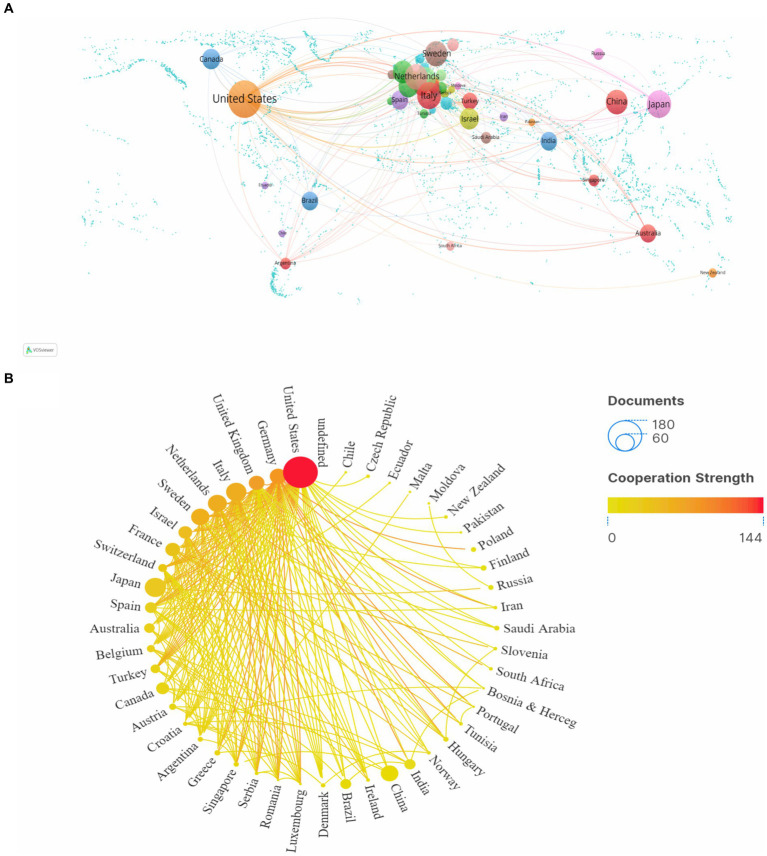
Analysis of the countries. **(A)** World the cooperation intensity map. **(B)** A circle diagram that evaluates international collaboration between clusters. Nodes represent countries, with node size indicating the number of publications, and lines showing the strength of collaboration.

A total of 49 countries and 221 institutions have participated in CTX research in the past 30 years. Table 1 displays the top 10 countries ranked by both publications and centrality. Betweenness centrality assesses a node’s significance in a network, where higher values indicate the node acts more frequently as a bridge in facilitating communication between other nodes (18). Nodes with centrality value >0.1 are deemed significant. Among the papers published in this research field, the United States made the greatest contribution, with 172 papers, far more than other countries. Japan, Italy, the Netherlands and Sweden ranked from 2 to 5. Figure 3 represents the CTX national collaboration network analysis. Figure 3A illustrates the geographical distribution of CTX publications, with a primary focus in North America and Western Europe. Figure 3B examines the international collaborations between clusters, where the size of the nodes reflects the number of publications and the lines denote the intensity of collaboration. As shown in Table 1 and Figure 3B, the United States is the core of the CTX collaboration network.

**Table 1:** The top 10 most publication countries.

Rank	Countries	Publications	Centrality	Countries	Centrality
1	USA	172	0.52	USA	0.52
2	Japan	64	0.16	Germany	0.28
3	Italy	59	0.08	UK	0.23
4	Netherlands	51	0.08	Spain	0.17
5	Sweden	48	0.06	Japan	0.16
6	China	44	0	Italy	0.08
7	Germany	40	0.28	Netherlands	0.08
8	UK	35	0.23	Greece	0.08
9	France	31	0.07	Russia	0.08
10	Israel	27	0.04	France	0.07

Based on CiteSpace LLR algorithm outcomes, the countries correspond to those of all authors.

**Figure 4:**
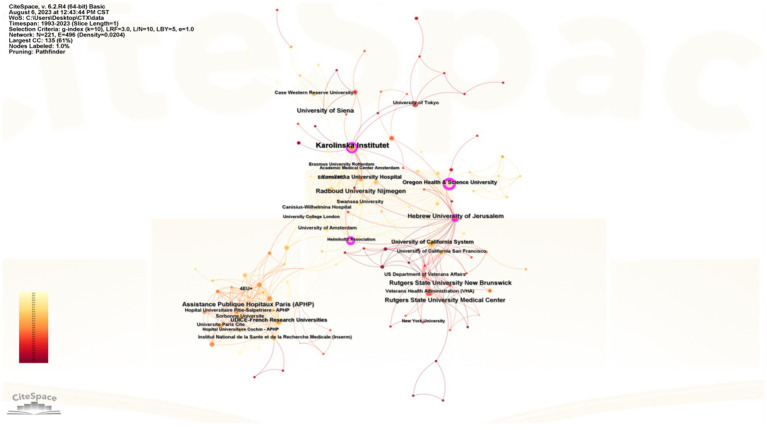
CiteSpace network map of institutions. Nodes represent institutions, with node size indicating publication volume, purple circles denote centrality >0.1, and lines signify the strength of collaboration.

Table 2 lists the top 5 most publication and top five most centrality institutions. 2 of the top 5 institutions are from the United States, and Karolinska Institute has the largest number of publications, with a total of 35 related papers. Figure 4 shows the institutional collaboration network, with purple circles highlighting entities with centrality values greater than 0.1, denoting their significance within the network. Oregon Health & Science University is the most centrality institution, which has some influence in the field of CTX.

**Table 2:** The top 5 most publication institutions.

Rank	Institutions	Country	Publications	Institutions	Country	Centrality
1	Karolinska Institute	Sweden	35	Oregon Health & Science University	USA	0.24
2	University of Siena	Italy	20	Helmholtz Association	Germany	0.20
3	Rutgers State University New Brunswick	USA	19	Hebrew University of Jerusalem	Israel	0.19
4	Rutgers State University Medical Center	USA	18	Karolinska Institute	Sweden	0.18
5	Radboud University Nijmegen	Netherlands	18	University of California System	USA	0.10

Based on CiteSpace LLR algorithm outcomes, the institutions correspond to those of all authors.

### 3.3 Analysis of authors and cited-authors

**Figure 5:**
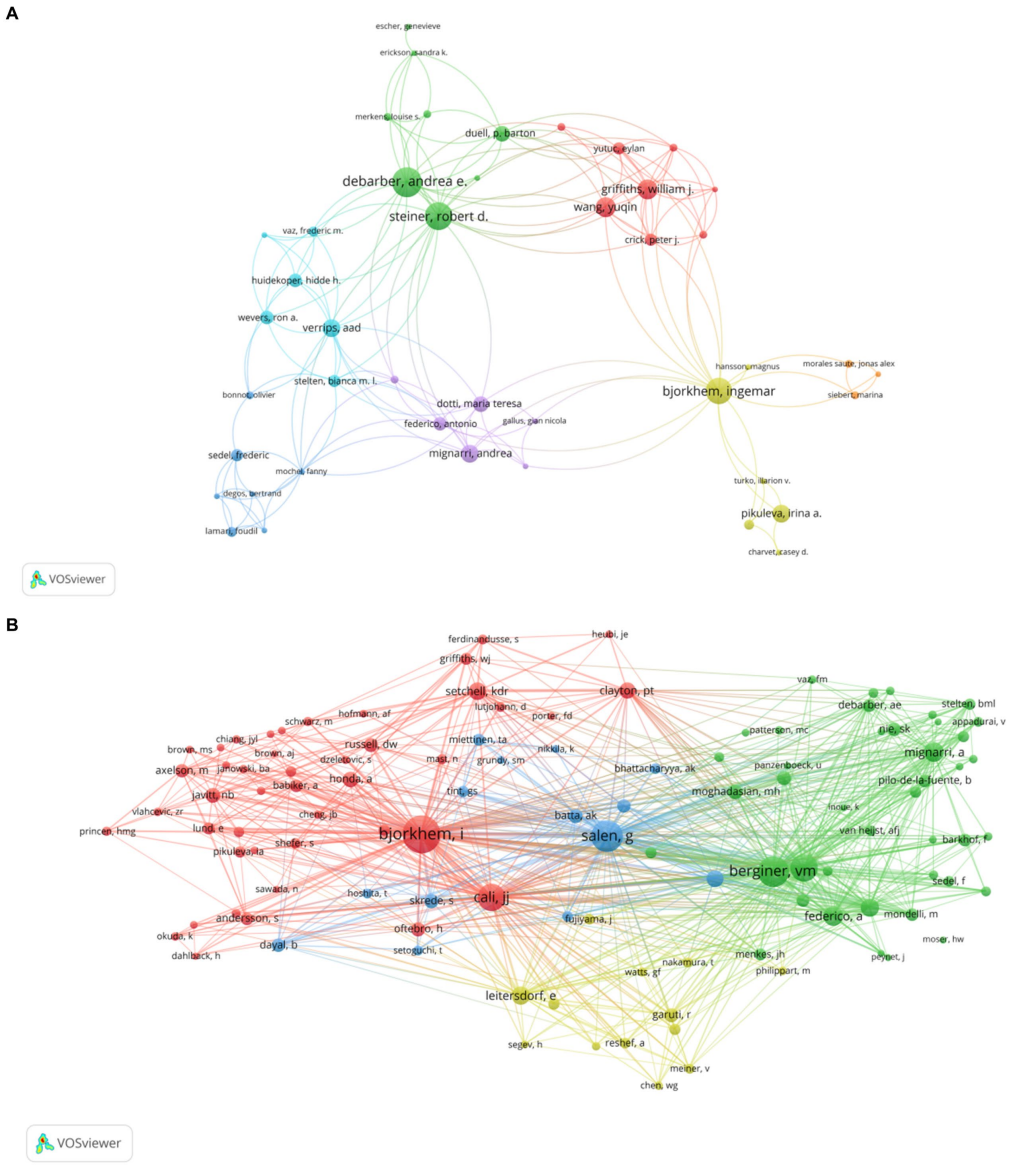
**(A)** Visualization of authors. **(B)** Visualization of cited authors. Nodes symbolize authors, in **A**, node size reflects the number of publications, in **B**, node size indicates citation frequency; different colors denote distinct clusters of collaborative relationships, and lines represent the strength of collaboration.

**Figure 6:**
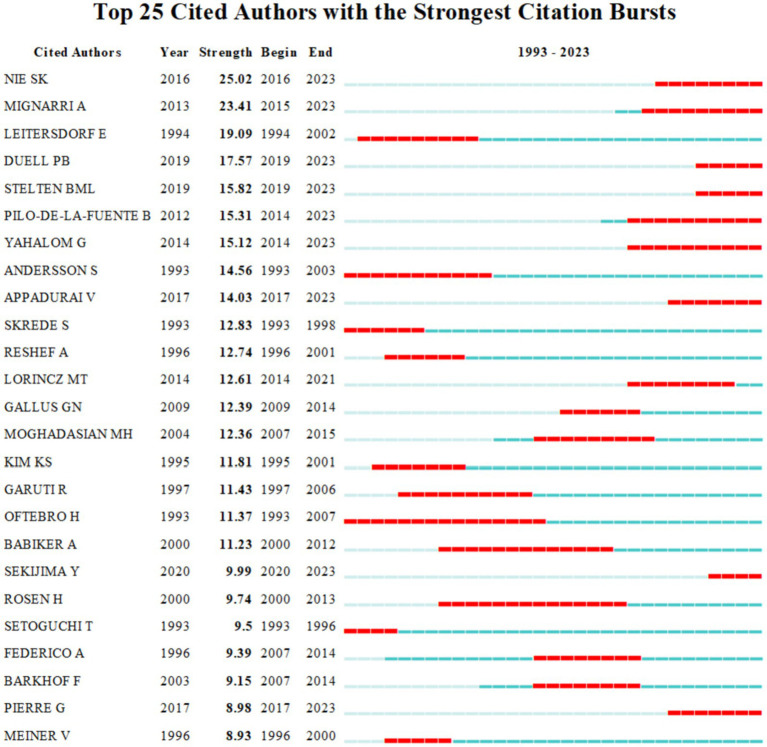
The top 25 cited authors with the strongest citation bursts. Strength indicates the intensity of citation bursts, with red denoting the duration of burst emergence.

Table 3 lists the top 5 authors with the most CTX publications and the most cited in CTX literature over the past three decades, primarily from Europe and the United States. The “co-citation analysis” feature identifies instances where two authors are cited together by other publications. CiteSpace software limits this analysis to the co-citations of the first author and counts multiple citations within the same publication once. The resulting co-citation network map reveals the academic collaborations within a research domain. Björkhem I. is the most prolific and cited author, with 23 publications and 254 citations. Figure 5 generated by VOSviewer, illustrates the author collaboration network. Figures 5A,B display the collaboration between authors and cited authors, with various colors indicating different clusters of collaborative relationships. These authors are at the central of distinct clusters, and collaborate closely. The burst detection analysis reflects hotspots emerging in field research, and when combined with timelines, it can clearly show the change trend of keyword, which effectively help the researchers in understanding shifts of the research focus. Among the top 25 cited authors in CTX publications from 1993 to 2023, Nie S. K. emerged as the author with the strongest burst of citations, while Oftebro H. had the longest duration of citation burst (Figure 6).

**Table 3:** The top 5 most publication authors and cited authors.

Rank	Author	Publications	Country	Cited-author	Cited frequency	Country
1	Björkhem I.	23	Sweden	Björkhem I.	254	Sweden
2	Salen G.	18	USA	Berginer V. M.	223	Israel
3	Federico A.	15	Italy	Cali J. J.	209	USA
4	Dotti M. T.	12	Italy	Salen G.	188	USA
5	Batta A. K.	11	USA	Verrips A.	164	Netherlands

Based on CiteSpace LLR algorithm outcomes, the authors represent all contributors to the publication. The table does not discriminate between productivity (number of papers published in filed) and citation numbers.

### 3.4 Analysis of journals and cited journals

**Figure 7:**
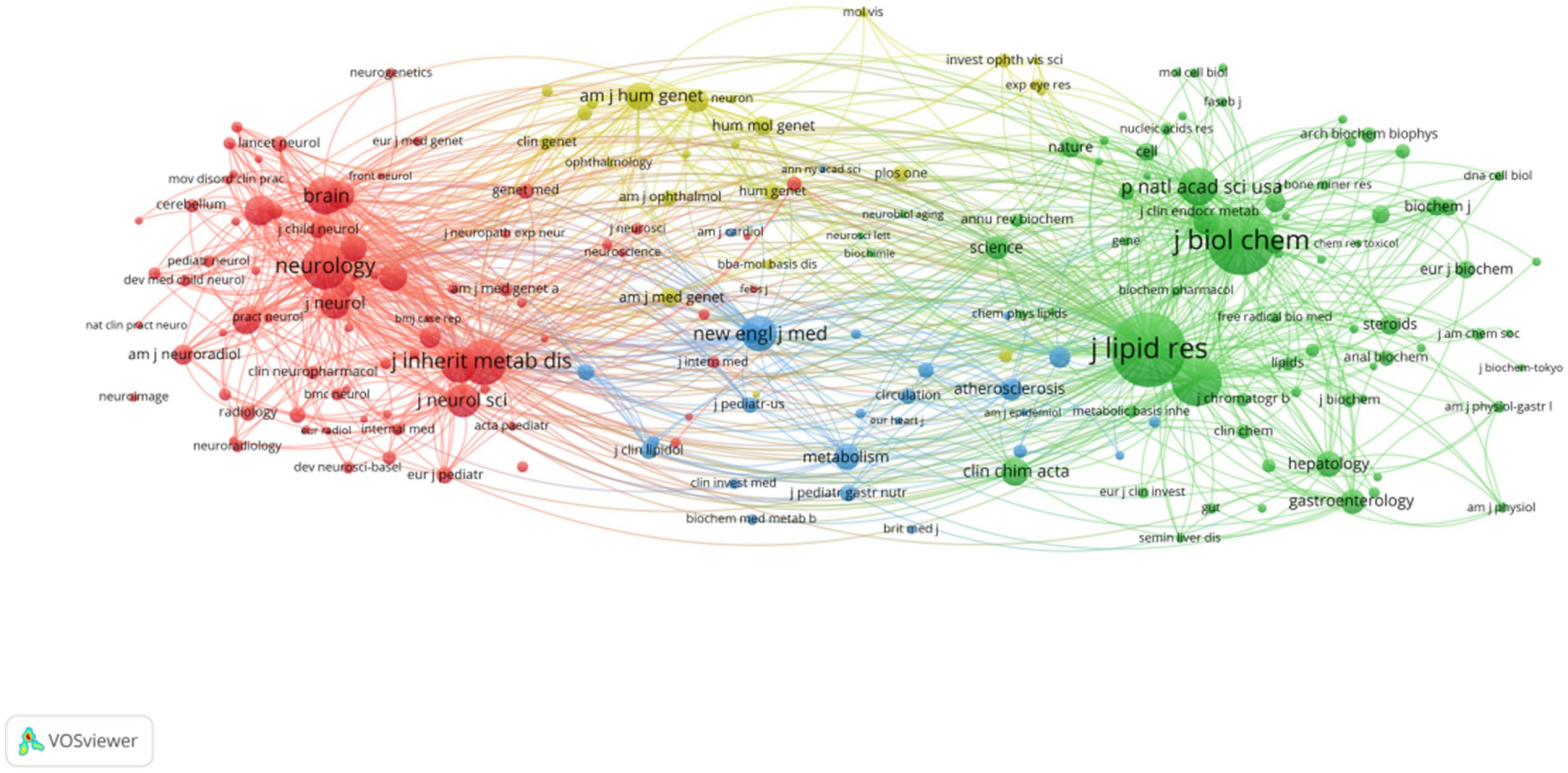
Visualization of cited journals. Nodes represent journals, with node size indicating citation frequency.

The top 5 publications and the top 5 cited journals are shown in Table 4. Table 4 shows that the top three prolific journals are Journal of Lipid Research, Journal of Inherited Metabolic Disease and Journal of the Neurological Sciences. In addition, Journal of Lipid Research is also the most frequently cited journal, followed by Journal of Biological Chemistry and Neurology. Journal of Lipid Research is the largest node in Figure 7. Combined with Table 4, it can be seen that its academic authority in the field of CTX research is better than that of other journals.

**Table 4:** The top 5 most publication journals and cited journals.

Rank	Journal	Publications	JCR quartile	Cited journal	Citations	JCR quartile
1	Journal of Lipid Research	43	Q1	Journal of Lipid Research	1,670	Q1
2	Journal of Inherited Metabolic Disease	21	Q2	Journal of Biological Chemistry	1,420	Q2
3	Journal of the Neurological Sciences	12	Q2	Journal of Clinical Investigation	756	Q1
4	Journal of Biological Chemistry	11	Q1	Neurology	645	Q1
5	Journal of Neurology	10	Q1	Journal of Inherited Metabolic Disease	624	Q2

### 3.5 Analysis of keywords

Keywords can reflect the hot spots and cutting-edge trends in the research field. Table 5 lists the top 10 high-frequency keywords, cerebrotendinous xanthomatosis (411), chenodeoxycholic acid (152), cholesterol (91), mutation (82), bile acid (71) in the top five, and all of them had a centrality > 0.1.

**Table 5:** Top 10 keywords with the highest frequency of occurrence.

Rank	Keyword	Occurrences	Centrality
1	cerebrotendinous xanthomatosis	411	0.35
2	chenodeoxycholic acid	152	0.16
3	mutation	91	0.14
4	cholesterol	82	0.19
5	bile acid	71	0.18
6	disease	51	0.12
7	diagnosis	49	0.03
8	metabolism	48	0.06
9	sterol 27 hydroxylase	46	0.12
10	cholestanol	45	0.09

**Figure 8:**
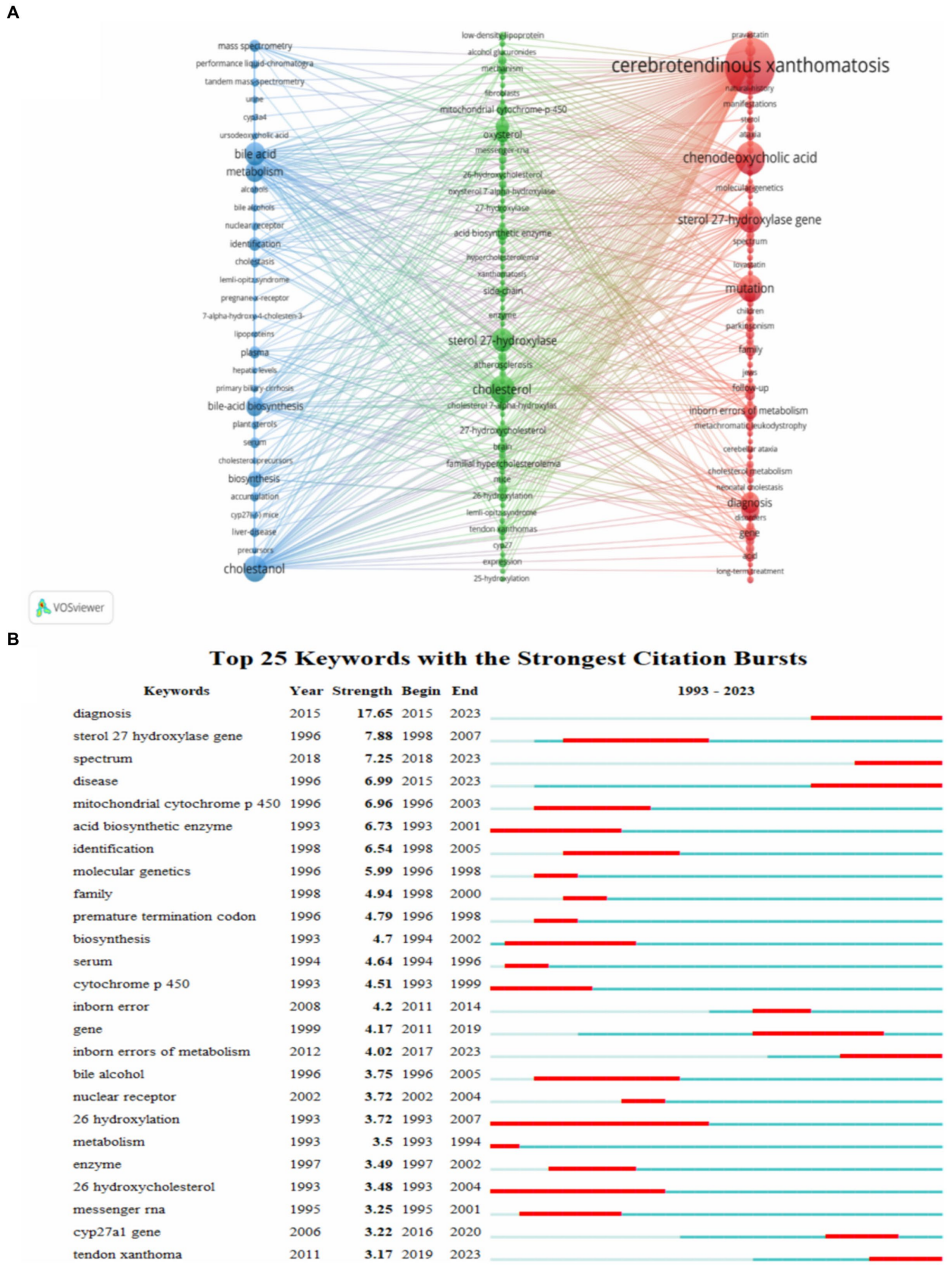
The analysis of keywords and cited references. **(A)** Visualization of keywords. **(B)** The top 25 keywords with the strongest citation bursts. Nodes denote keywords, with node size reflecting co-occurrence frequency. Strength indicates the intensity of citation bursts, with red signifying the duration of burst emergence.

The analysis of keyword co-occurrence by VOSviewer showed that the keywords formed three clusters as shown in Figure 8A. The first cluster (red) mainly includes the diagnosis and treatment of CTX such as diagnosis disease follow-up therapy. The second cluster (green) mainly explores the pathogenesis of CTX such as cholesterol sterol 27-hydroxylase bile acid biosynthesis oxysterol. The third cluster (blue) mainly focused on the prognosis of CTX such as metabolism primary biliary-cirrhosis neonatal cholestasis liver-disease.

Keywords were detected and analyzed for bursts using CiteSpace, Figure 8B shows the keywords in the top 25 of burst intensity. Diagnosis ranked first in burst intensity, followed by sterol 27 hydroxylase gene (7.88) and acid biosynthetic enzyme (7.25). 26 hydroxylation is a keyword that burst lasts for a long time. In recent years, the research hotspots for bursts have been diagnosis, disease, *CYP27A1* gene, inborn error metabolism and spectrum.

Over the past three decades research has greatly enhanced our understanding of CTX which can be outlined as follows: (1) Insight into the genetic landscape with the identification of several new and rare *CYP27A1* gene mutations contributing to the recognition of genetic variability in CTX (19). (2) Detailed exploration of clinical symptoms where early detection and intervention have been shown to notably improve patient prognoses (20, 21). (3) Advancements in diagnostic approaches with the rise in levels of 7alpha-hydroxy-4-cholesten-3-one and cholesterol precursors confirmed as significant biomarkers for CTX (22). The broad use of imaging techniques such as MRI to detect the deposition of cholesterol and lipids in the brain (23).

## 4 Discussion

### 4.1 General information

From the curve fitting results in Figure 1, it can be seen that although the annual number of papers published fluctuates greatly, the cumulative number of papers shows a rapid growth trend as a whole. From each viewpoint of time, the number of published articles was unstable from 1999 to 2005, and dropped sharply to 8 in 2005, which indicated that the study of CTX was still in its infancy and did not attract the attention of scholars at that time. However, the curve has shown an overall upward trend since 2005 and reached a peak in 2021.

From the perspective of countries and institutions, the United States is the main contributor, and it is the country with the largest number of publications and the highest centrality. Although the number of German publications ranks 7, its centrality was 2nd, which indicated that its academic status and influence in the field of CTX are better than others. As for China, although it has 44 publications, its centrality was 0, which showed that China has little cooperation with other countries and needs to strengthen effective academic exchanges. Most of the top 5 institutions were located in North America and Europe, and Oregon Health & Science University and Helmholtz Association were the top 2 centrality institutions, which showed that they are playing an important role in the field of CTX research and their international academic influence has been further strengthen.

Regarding author productivity, Björkhem I. has published the most works in the last three decades, with Salen G. and Federico A. ranking second and third, respectively. In addition, Björkhem I. stands as the author with the highest number of citations in publications, achieving considerable progress in the biochemistry of CTX. He found that sterol 27-hydroxylase is a key enzyme in the conversion of cholesterol to 27-oxygenated steroids, and that loss of sterol 27-hydroxylase induces CTX and is associated with an early risk of atherosclerosis (24–26). Salen G. and Berginer V. M. have made groundbreaking contributions to the identification and treatment of CTX. Salen and his team had confirmed that CDCA is the most effective treatment. There is a significant increase in the risk of osteoporosis and fracture in patients with CTX (27, 28). Berginer and his team contributed to the accurate diagnosis of CTX by investigating the genetic architecture and traits of prevalent CTX mutation alleles among Moroccan Jews (29). Among the top 25 cited authors, Nie S. K. and his team indicate that increased cholesterol levels in CTX patients may lead to degeneration in the nigrostriatal dopaminergic pathway, potentially exacerbating the progression of Parkinson’s syndrome (30). Oftebro H. found that the metabolic defect in CTX is due to a complete absence of mitochondrial C27-steroid 26-hydroxylase (6).

With regard to journals, it can be seen from Table 4 that Journal of Lipid Research is the journal with the most output and citations, which shows that the journal has made great contributions in the field of CTX research. The journals listed in Table 4 belong to the Q1 or Q2, which indicated that these journals have a high academic status in CTX research and are supported by the majority of scholars.

### 4.2 The hotspots and frontiers

Based on keyword co-occurrence, clustering, burst detection analysis and burst detection analysis of references, we have established the research hotspots of CTX: pathogenesis, diagnosis and treatment.

#### 4.2.1 Pathogenesis

CTX is caused by a mutation in the *CYP27A1* gene that encodes sterol 27-hydroxylase, a key enzyme in the bile acid biosynthesis pathway (31, 32). The classical pathway is initiated by cholesterol 7 α-hydroxylation and catalyzed by rate-limiting enzyme cholesterol 7 α-hydroxylase. The alternative pathway is initiated by sterol 27-hydroxylase catalyzed 27-hydroxylation of cholesterol (33). The decrease of sterol 27-hydroxylase activity leads to the impairment of bile acid synthesis in classical and alternative pathways, resulting in the decrease of bile acid (especially CDCA) production and the decrease of bile acid production to a lesser extent. The loss of negative feedback of CDCA on cholesterol 7 α-hydroxylase accelerates these metabolic abnormalities, resulting in an increase in the level of bile acid intermediate 7alpha-hydroxy-4-cholesten-3-one, a precursor of cholesterol and bile alcohol (34).

#### 4.2.2 Diagnosis

The diagnosis of CTX usually involves clinical observations, biochemical assessments, and genetic evaluations, with neuroimaging acting as a supportive diagnostic criterion. The most notable neuroradiological features are the presence of signal hyperintensities observed in T2-weighted and/or FLAIR imaging, particularly in the dentate nuclei and the surrounding cerebellar white matter (35, 36). Early diagnosis of CTX is crucial, as initiating treatment in the early stages can significantly improve symptoms and slow the deterioration of neurological functions (37). However, delayed diagnosis of CTX is common. Koyama et al. (33) proposed a new diagnostic criterion, which can be diagnosed as CTX only when there is at least one clinical symptom associated with CTX and the serum cholestanol is elevated (≥4.5 μg/mL, mean ± SD: 2.35 ± 0.73 μg/mL). Genetic testing is essential, as known mutations in the *CYP7A1* gene have been identified as triggers for CTX (38, 39).

#### 4.2.3 Treatment

The most effective treatment is chenodeoxycholic acid therapy (2, 40). It operates by suppressing the synthesis of bile acids via a negative feedback pathway, effectively hindering the buildup of cholestanol (41). Studies have demonstrated that CDCA is not only effective in the long-term treatment of CTX but also maintains a favorable safety profile (42).

### 4.3 Limitation

There are some limitations to our study. To begin with, we exclusively utilized the WoSCC database for our search. Moreover, we only included publications that were in English-language publications and categorized them as articles or reviews. Lastly, the basic functionalities of CiteSpace and VOSviewer were constrained, enabling us to analyze citation data for a maximum duration of 30 years.

## 5 Conclusion

CTX literature from 1993 to 2023 were summarized and visually analyzed. The results showed that although the number of articles published every year fluctuates greatly, the depth and breadth of the research are constantly expanding. Although more and more countries, institutions and scholars have focused on CTX, the pathogenesis, diagnosis and treatment of CTX are still difficult problems to be solved urgently. Therefore, more efficient and accurate early diagnosis and treatment will be the focus of future research.

## Data availability statement

The raw data supporting the conclusions of this article will be made available by the authors, without undue reservation.

## Ethics statement

Ethical review and approval was not required for this study in accordance with the local legislation and institutional requirements.

## Author contributions

FL: Writing – original draft, Writing – review & editing. YD: Data curation, Visualization, Writing – review & editing. SZ: Software, Writing – review & editing. JD: Supervision, Writing – review & editing. BY: Conceptualization, Writing – review & editing.

## Footnotes

^1^ https://bibliometric.com/
