# Corrigendum: Frontier and hotspot evolution in cerebrotendinous xanthomatosis: a bibliometric analysis from 1993 to 2023

**DOI:** 10.3389/fneur.2024.1484618

**Published:** 2024-09-10

**Authors:** Fei Luo, Yali Ding, Shanyun Zhang, Juanjuan Diao, Bin Yuan

**Affiliations:** ^1^Department of Pediatrics, Affiliated Hospital of Nanjing University of Chinese Medicine, Nanjing, China; ^2^Department of Pediatrics, Nanjing Gaochun Traditional Chinese Medicine Hospital, Nanjing, China; ^3^Department of Pediatrics, Haining Hospital of Chinese Medicine, Haining, China; ^4^Department of Pediatrics, Affiliated Hospital of Shandong University of Traditional Chinese Medicine, Jinan, China

**Keywords:** bibliometric analysis, cerebrotendinous xanthomatosis, CiteSpace, VOSviewer, Web of Science

In the published article, there was an error in the author list. Equal author contributions were not attributed to Yali Ding and Shanyun Zhang. The correct equal author contributions appears below.

Fei Luo^1†^, Yali Ding^2†^, Shanyun Zhang^3†^, Juanjuan Diao^4^ and Bin Yuan^1*^

^†^These authors contributed equally to this work and share first authorship.

The authors apologize for this error and state that this does not change the scientific conclusions of the article in any way. The original article has been updated.

